# Implementation challenges and opportunities for HIV Treatment as Prevention (TasP) among young men in Vancouver, Canada: a qualitative study

**DOI:** 10.1186/s12889-016-2943-y

**Published:** 2016-03-15

**Authors:** Rod Knight, Will Small, Kim Thomson, Mark Gilbert, Jean Shoveller

**Affiliations:** Faculty of Health Sciences, Simon Fraser University, Burnaby, Canada; British Columbia Centre for Excellence in HIV/AIDS, Vancouver, Canada; School of Population and Public Health, University of British Columbia, 2206 East Mall, Vancouver, British Columbia V6T 1Z3 Canada; Ontario HIV Treatment Network, Toronto, Canada

**Keywords:** Treatment as prevention, HIV/AIDS, Young men, Canada

## Abstract

**Background:**

Despite evidence supporting the preventative potential of HIV Treatment as Prevention (TasP), scientific experts and community stakeholders have suggested that the success of TasP at the population level will require overcoming a set of complex and population-specific implementation challenges. For example, the factors that might influence decisions to initiate ‘early’ treatment have yet to be thoroughly understood; neither have questions about the factors that enhance or impede their ability to achieve long-term adherence to ARVs or the social norms regarding various treatment regimens been examined in detail. This knowledge gap may hamper opportunities to effectively develop public health practices that are informed by the various challenges and opportunities related to TasP implementation and scale up.

**Methods:**

Drawing on 50 in-depth, individual interviews with young men ages 18–24 in Vancouver, Canada, this study examines young men’s perspectives regarding factors that might affect their engagement with TasP.

**Results:**

While findings from the current study indicate young men generally have a high receptiveness to TasP, our findings also identify key social and structural forces that will warrant ongoing consideration for TasP implementation. For example, participants described how an enhanced awareness regarding treatment (including awareness of the universal availability of treatment in Vancouver) would be a necessary, but not sufficient, condition to decide to endorse TasP. Their decisions about engaging in HIV care in the context of TasP (e.g., HIV testing, treatment initiation, long-term adherence) also appear to be contingent on their ability to negotiate or ‘balance’ the risks and benefits to themselves and others. The findings also offer insight into the complex and sometimes controversial narratives that continue to emerge regarding risk compensation practices in the context of TasP.

**Conclusion:**

Based on the results of this study, we identify several areas that hold promise for informing the effective scale up of TasP, including new information regarding implementation adaptation strategies.

## Background

A growing number of clinical trials, cohort studies and mathematical modeling analyses indicate that antiretroviral (ARV) treatment is effective in reducing the onward transmission of HIV from seropositive to seronegative individuals [1–5]. As a result, regional and global HIV prevention efforts are increasingly relying upon HIV Treatment as Prevention (TasP) approaches as a key component of broader efforts to address the HIV epidemic, as evidenced by the UNAIDS’ [6] recent adoption of the ‘90-90-90’ testing, treatment and viral load suppression targets. Despite evidence supporting the preventative potential of TasP, scientific experts and community stakeholders have suggested that the success of TasP at the population level will require overcoming a set of complex and population-specific implementation challenges.

### Treatment as prevention

The role of TasP within the rapidly transforming ARV-based prevention arena has had a significant impact in re-shaping the broader HIV continuum of care. Today, TasP is being implemented as a means to reduce sexual- and injection-related transmission of HIV among a variety of populations. Within the evidence in this area, a landmark clinical trial-HPTN 052-indicated that ‘early’ initiation of ARVs can reduce the transmission of the HIV virus among heterosexual serodiscordant couples by up to 96 % compared to couples who initiate therapy at previously recommended World Health Organization thresholds (CD4 count < 250 cells/mm^3^) [5]. More recently, the PARTNER Study (an investigation of HIV transmission through vaginal and anal condomless sex between serodiscordant heterosexual and MSM partners) reported that, among couples initiating ARV treatment, none had transmitted the virus to their partners [7]. Given these promising clinical and epidemiological findings, treatment is now widely accepted as having both individual and community health benefits in that it can prevent both HIV-related morbidity (e.g., progression to AIDS), as well as the onward transmission of HIV infection [8, 9].

### ‘Fast-tracking’ the response to HIV/AIDS with TasP

In 2014 the UNAIDS called for a global scale-up of TasP and efforts to meet the following ‘90-90-90’ targets by 2020: (1) 90 % of all people living with HIV will know their HIV status; (2) 90 % of all people diagnosed with HIV infection will receive antiretroviral therapy (ART), that includes the use of ARVs; and (3) 90 % of all people receiving ART will achieve viral suppression. These commitments established a set of highly ambitious targets regarding the scale-up of TasP that, as indicated in the ‘90-90-90’ target guidelines, require various global and local settings to ‘*tailor approaches to address the unique challenges in diverse settings and populations*’ [6].

Since 2010, Vancouver’s regional health authority has implemented a voluntary routine offer model for HIV testing in primary and acute care settings, aiming to increase HIV testing rates by embedding testing within routine care. Funding and policy commitments have also supported universal availability of ARVs for HIV-positive individuals, ‘earlier’ initiation of ARVs (i.e., as soon as possible following seroconversion and regardless of an individual’s CD4 count), and enhanced efforts to treat all clinically eligible HIV-positive persons [10]. As such, Vancouver provides an interesting implementation context within which to examine factors that can influence the scalability of TasP among a variety of key-affected populations.

### Young men and HIV care

Scaling up TasP programmes and service delivery practices may present a particularly salient set of implementation issues among young men-a group with high and rising rates of HIV in addition to disproportionately low levels of engagement with health care seeking behaviour generally and HIV care specifically (e.g., compared to older men and/or women). In 2012, for example, HIV incidence rates for men between the ages of 20–24 and 25–29 in BC were significantly higher than the provincial average at 7.7 and 22.0 cases per 100,000 (compared to the provincial average of 5.2 per 100,000) [11]. Moreover, new HIV diagnoses in Vancouver have been increasing among younger cohorts of MSM (born 1980–1999), as compared with decreasing numbers of new HIV diagnoses among MSM cohorts born pre-1980 [12]. As a result, HIV transmission in BC has been described as a ‘re-emerging epidemic’ among young men, particularly in marginalized subgroups of men including MSM and men who inject drugs [12].

Previous work has shown how socio-cultural influences affect young men’s (non) participation in HIV testing [13–16], particularly through social norms regarding masculinity, stigma and wait times [15, 17]. Young men also face challenges when accessing sexual health services, including barriers to effective communication with health professionals, that can be exacerbated through enactments of hegemonic masculinity [13, 14, 18–20]. Furthermore, theories of gender relations, masculinities and men’s health behaviour suggest that men’s health experiences are influenced by the wider set of social relations [21–24] that vary across other aspects of the social hierarchy (e.g., socio-economic status). In general, men are less likely than women to seek care from health care professionals and more likely to engage in medium- to long-term self-treatment strategies, as well as position ‘help-seeking’ behaviour as potentially emasculating [22, 23, 25, 26].

While there is a growing empirical and theoretical literature related to young men’s health-related practices regarding their (non)participation with HIV testing, far less is known about how young men perceive other levels of the HIV continuum of care, particularly in the rapidly evolving context of TasP. Previous research into various levels of the HIV cascade of care, however, have been extremely helpful at identifying how structural influences, including HIV stigma, can have vast implications for particular experiences with the HIV cascade of care that hold relevance for the imperatives of TasP (e.g., barriers and facilitators to: HIV testing patterns; risk-reduction practices; capacity for long-term adherence to ART) [27]. With respect to TasP, a recent review [28] of empirical literature investigating the acceptability of ARV-based prevention strategies (specifically, TasP and Pre-Exposure Prophylaxis-PrEP) found that only three of 33 relevant articles focused on TasP. Furthermore, among the three TasP-related articles [29–31], none focused on the needs or perceptions of young men specifically.

There is currently a dearth of information related to how young men might respond to or perceive the evolving HIV continuum of care in the context of TasP and given the universal availability of treatment in some settings, including what TasP means for evolving ‘risk landscapes’ and testing practices of young men who are at risk of contracting HIV, as well as those who may not be aware of their serostatus. Moreover, the factors that might influence young men’s decisions to initiate ‘early’ treatment have yet to be thoroughly understood; neither have questions about the factors that enhance or impede their ability to achieve long-term adherence to ARVs or the social norms regarding various treatment regimens been examined in detail. To date, these and other issues, including young men’s perspectives on other potential unanticipated consequences that may arise in the context of TasP (e.g., risk compensation practices, such as reduced rates of sustained condom and/or sterile syringe use), remain largely unknown. This knowledge gap may hamper opportunities to effectively develop public health practices that are informed by the various challenges and opportunities related to TasP implementation and scale up among young men who are at risk for HIV and/or may not be aware of their serostatus.

### Purpose

The purpose of this study is to examine the knowledge, attitudes and normative understandings of young men who are at risk for HIV acquisition, including those who may not be aware of their serostatus, regarding the testing, treatment and long-term suppression imperatives of TasP. To do so, we draw on data gathered through semi-structured, in-depth individual interviews with 50 young men (ages 18–24) in Vancouver, Canada.

## Methods

For the reader, it is helpful to consider our motivations for conducting this analysis. The current analysis was conducted as a part of a larger program of research identifying the structural and socio-cultural determinants of young men’s sexual health, with particular attention on the ethical and implementation factors associated with Vancouver’s evolving HIV intervention ‘landscape’. Our analysis is grounded within a critical realist perspective [32, 33] that hypothesizes that socio-structural features of implementation context are dialectically interrelated with both the outcomes of an intervention (in this case, TasP) and the experiences of the intervention ‘targets’ (in this case, both young men who do and do not know their serostatus, and/or are at risk of HIV). For example, we aim to identify how features of contemporary socio-cultural contexts (e.g., norms about young people’s sexual health practices) influence young men’s perspectives and experiences regarding the testing, treatment and prevention imperatives of TasP.

### Recruitment and data collection

Data were collected between June and November 2013 in Vancouver, British Columbia (BC) in Canada, which provides an ideal setting to examine perspectives about TasP. We drew on a stratified purposeful approach to sampling in order to capture variation within and across various sub-groups of young men (e.g., various lived experiences; social identities; behavioural, social and structural HIV risk profiles). In total, fifty young men ages 18–24 were recruited to participate in the study through advertisements at clinical sites (e.g., posters at youth sexual health clinics) and non-clinical settings (e.g., youth centers; bus stops), as well as online (e.g., Facebook advertisements; Craigslist). Participants also were recruited from the At-Risk Youth Study (ARYS), a prospective cohort of Vancouver youth who are or have previously been street-involved and used illicit drugs (other than marijuana) (see Wood et al. [34] for more information on the ARYS cohort). Eligibility criteria include: ages 18–24; ability to speak and understand English; identify as a man (including cisgender and/or trans* identified men); currently sexually active or have been sexually active previously.

Participants completed an informed consent form and a 9-item socio-demographic questionnaire, prior to participating in an in-depth, semi-structured, individual interview (see Table 1 for additional information on the questions and probes used during our interviews to discuss some of the tenets of TasP). All interviews were audio-recorded and were conducted within our team research offices. Participants were offered the choice to be interviewed by a male interviewer (co-author RK) or a female interviewer (a research assistant), though none expressed a preference. Interviews lasted 1 to 1.5 h in duration and were conducted by interviewers with training in qualitative health research methods, as well as extensive experience conducting interviews with young men regarding sexual health. Participants were asked to describe their thoughts regarding HIV treatment (e.g., availability; effectiveness) in Vancouver (and elsewhere). They also were asked to describe their perceptions regarding the effectiveness of HIV treatment in terms of the prognoses for HIV-positive individuals as well as their opinions regarding the capacity of TasP to protect seronegative partners (e.g., partners with whom they share drugs; sex partners). Participants were provided with a description of TasP and asked to describe the extent to which they felt these approaches were ‘fair’ and ‘justifiable’. Participants also were asked to explain whether or not they perceived TasP to be something they might consider for themselves (i.e., would they choose to initiate treatment in the event of an HIV positive diagnosis for either or both treatment and prevention reasons?).

**Table 1:** Questions and probes to specifically discuss TasP during the interviews

Questions	Probes
Some people have told us that because there are more advanced HIV treatment opportunities, some of the concerns related to HIV are no longer as important.	
a. What do you know about HIV treatment? b. Providing treatment to people who are HIV+ can also make them less infectious-in other words, if somebody who is HIV+ is receiving treatment, they will not be as likely to give HIV to someone else. This has been referred to as ‘treatment as prevention’, because the treatment is offered to patients to prevent HIV transmission, regardless of whether or not the individual is at a stage of HIV infection that requires treatment for their individual benefit (e.g., so they don’t become sick). What are your thoughts on offering treatment to individuals who are HIV+ in order to prevent them from transmitting HIV?	• Tell me how the availability of HIV treatment might influence your decision to go for HIV testing? How might the availability of treatment affect the testing decisions of your friends (guys/girls)? • How acceptable do you think it would be if you were HIV+ and you were offered treatment so that you would not be infectious, rather than for your own health-related concerns? • Some have argued that it might be unfair to treat an HIV-infected person in order to prevent them from infecting others because it places another demand on people who might be very vulnerable people (e.g., poor people; people who use injection drugs). Can you tell me what your thoughts are on the ‘fairness’ of this approach?

We did not ask participants to disclose their HIV status, a decision we arrived at only after considerable discussion and debate within our team. In the end, we chose not to ask interview participants to disclose their status for the following reasons. Firstly, the legal system in Canada currently adopts a “criminalization” approach. A 2012 ruling by the Supreme Court of Canada has upheld the criminalization of HIV non-disclosure except where *both* a condom is used and the person has a ‘low’ HIV viral load. A conviction of aggravated sexual assault could result if these requirements are not followed. Bearing all this in mind, we thought that it was not warranted from an ethical perspective to ask study participants to disclose their serostatus to us. Instead, we have provided context regarding various aspects of HIV-related risk at the individual level (e.g., use of condoms; injection drug use) as well as at the structural level (e.g., recent experiences with being street-entrenched or homeless).

Participants received a CDN$25 honorarium to compensate them for their participation. Ethics approval was obtained from the University of British Columbia’s Behavioural Research Ethics Board (#H12-01936).

### Data analysis

Interviews were transcribed and accuracy checked and then uploaded to Nvivo10. First, co-authors RK and KT read and re-read the transcripts, assigning initial codes that were then grouped thematically. Next, we used an open-coding approach in which coding was first organized into ‘trees’ to group the codes thematically. The emergent thematic codes focused on capturing the micro- (e.g., previous experiences), meso- (e.g., interpersonal relations) and macro (e.g., socio-cultural) factors that shape men’s opinions and behaviour related to HIV, with a specific focus on ARV treatment initiation and long-term adherence (e.g., successes, challenges). Discrepancies between codes and coders were resolved through discussion and re-visiting the raw data at coding during team meetings. Next, each thematic was explored further by asking three key analytic questions: (a) How do men perceive HIV care in Vancouver in the context of universally available treatment, and how might this influence their engagement with TasP programmes and service delivery practices?; (b) What are the factors that influence men’s perceptions regarding HIV treatment and TasP?; and (c) What do participants raise as important challenges or opportunities for young men’s engagement with TasP? Thus, as we conducted our thematic analysis, we employed both inductive and deductive approaches by continually comparing our emergent themes with the existing literature in this area with our empirical data [35].

## Results

### Study participants

A total of 50 young men completed interviews. Participants ranged in age from 18 to 24 (mean age: 21.7). Thirty-two percent (*n* = 16) of participants identified as gay, bisexual or Two-Spirit, 68 % (*n* = 34) as straight. Forty-eight percent (*n* = 24) were recruited from the ARYS cohort, with the remaining 52 % (*n* = 26) recruited either through online advertising or posters. Table 2 provides additional socio-demographic information of our sample.

**Table 2:** Socio-demographic characteristics of study sample

Ethnicity	*(n)*	(%)
Aboriginal	6	12
African-Canadian	1	2
Euro-Canadian	26	52
Latin	2	4
South East Asian	7	14
Middle Eastern	1	2
Other	7	14
Living arrangement		
With parents	9	18
With friends or partner	22	44
Alone	7	14
In a shelter or on the street	11	22
In a recovery house	1	2
Sexual Orientation		
Bisexual	8	16
Gay	7	14
Heterosexual/straight	34	68
Two-Spirit	1	2
Gender Identity		
Transgender man	1	2
Cisgender man	49	98
Recruitment mechanism		
Online advertising	23	46
Posters	2	4
ARYS study (see recruitment details for information on the ARYS study)	24	48
Other	1	2

### Overview of findings

We report our findings using two thematic categories regarding the participants’ perceptions of TasP: (1) Reflections about treatment and treatment initiation in the context of TasP; and (2) Perceived challenges and opportunities regarding long-term viral load suppression. Quotations from participants’ transcripts are presented to illustrate the various themes that arose during our interviews and related to each thematic. Each quotation is preceded by a short description of each participant’s socio-demographic profile and a researcher-assigned numeric code follows each quote.

### Reflections about treatment and treatment initiation in the context of TasP

Our interviews began by asking participants to describe how their understandings of contemporary HIV treatment regimens might influence their engagement with HIV care. Within this conversation, some participants explained that they had limited knowledge regarding HIV treatment and asked our interviewers for some additional information. We explained to the participants that, when effectively treated and diagnosed early, HIV seropositive individuals can expect a near normal quality of life and longevity. Upon reflecting on this information, several participants indicated that knowing more about the availability and effectiveness of HIV treatment might make them more likely to access HIV testing services. For example, a 24-year-old straight man described:

*I think knowing what it would be like if I tested positive would make me more prone to get tested, ‘cause, like, I don’t know how justified this is, but I had this idea that the prognosis is pretty bad. Maybe that’s just ignorance, I don’t know. But I think if people knew what happens next more, it would make testing more easy to decide to do.* (#020)

After reflecting on how available and highly effective HIV treatment might influence their HIV testing practices, several participants expressed concern that, despite the effectiveness of treatment, there would likely be ‘insurmountable’ economic barriers to acquiring treatment that they would not be able to overcome – and these concerns were particularly salient among men experiencing multiple and intersecting forms of disadvantage (e.g., living on the streets; addiction issues). For example, after being informed that treatment can greatly improve an HIV seropositive individual’s quality and length of life, a 22-year-old bisexual man and a 20-year-old straight man expressed concern:

*But I don’t know if that* [information about HIV treatment and prognoses] *makes me any less concerned about certain aspects of the disease just because I also know that it’s* [treatment] *expensive, and that definitely plays into me, ‘cause I know that if I needed to afford expensive medication, I just couldn’t right now.* (#018)

*You’re still fucked* [in the event of an HIV diagnosis]*. I don’t even know if it’s covered by MSP* [Medical Services Plan, the provincial health care plan in the province of British Columbia]*. I highly doubt it. So if you’re on welfare and you have HIV, I think you’re pretty fucking screwed. And I would not want to have to figure it out, either.* (#041)

During this part of our interviews, we explained that HIV treatment is universally available free-of-charge through the medical services plan in BC to clinically eligible patients. We also described to participants that there are preventative benefits associated with HIV treatment by explaining that HIV seropositive individuals who achieve viral suppression are less likely to transmit HIV to their uninfected partners. Upon reflecting on this information, most participants were eager to learn more about a variety of aspects of HIV treatment. For example, when we asked them to consider the different pieces of information that they would need to know in order to inform their decision about treatment initiation, the majority of participants described that they would want to learn more about the various potential side effects associated with HIV treatment. For instance, a 23-year-old straight participant explained how his decision-making about treatment initiation would require a careful balance of the health benefits versus the potential side effects associated with drug toxicity:

Participant: *It* [initiating ARVs] *all depends on the side effects of the treatment. If the side effects were really serious, I probably would not want to do it, because side effects from drugs, from treatment like that, could be very serious. So, yeah, I probably would not do it if the side effects were too serious.*

*Interviewer: Okay. And, if the side effects weren’t too bad?*

*Participant: I would be very glad to do it so I don’t spread it to other people.* (#028)

At this point in our interviews, we provided participants with some additional information regarding HIV treatment, including a description of how treatment is now generally considered to be safe if treatment regimes are effectively followed. As participants reflected on HIV treatment and whether they might consider initiating treatment, two dominant themes emerged from their narratives about why they would consider initiating treatment immediately following an HIV diagnosis. First, participants described how preventing future harm (i.e., transmitting HIV) to their sex partners would represent an important consideration. For example, one 22-year-old bisexual participant described:

*Personally, I’d go on that sort of treatment just to make sure that it is safe for my partners.* (#018)

The preventative benefits of treatment were also positioned as constituting a “public safety” measure in the participants’ narratives about HIV treatment. For example, another 22-year-old bisexual participant described:

*If the medicine is destructive on the body then there are certain compromises that can’t be made. But from a point of public safety, I think it is important for somebody to take it if they’re gonna be putting themselves at risk of spreading it.* (#007)

Similarly, among a small sub-set of participants, some elaborated how treatment would also be a beneficial means to protect the partners with whom they exchange or share injection drug equipment. For example, a 24-year-old straight man who had previously injected drugs described how he would expect others who seroconvert and inject drugs to initiate treatment in order to help prevent the likelihood of onward transmission through contaminated equipment:

*I couldn’t imagine being like, “No, I don’t want to go through treatment,” and just, like then being completely sloppish about disposing of anything with blood on it or like needles. I just don’t know why someone would want to run the risk of infecting someone. Like, why wouldn’t you want to get treatment and just try to nip it in the bud as quick as you can?* (#031)

We also asked them to consider how they would want to be offered treatment in the event of an HIV diagnosis. Some of the participants expressed concern that clinical communication strategies would need to be tailored in a way that transparently delineates the various risks and benefits associated with ‘early’ treatment initiation. For example, one 23-year-old straight man explained:

*I think if it’s* [clinical recommendations regarding ARV initiation] *sort of proposed in the form of a question or as a sort of an option amongst several, right? Then I think those sorts of things continue to respect the autonomy of other people. Whereas if it’s kind of, you know, strongly recommended or, you know, bordering on coercive, then I would question the legitimacy of that kind of approach.* (#013)

Some participants described how seropositive individuals could feel ‘targeted’ within clinical encounters if they perceived that their clinician was emphasizing public health benefits (e.g., prevention of onward transmission) over clinical concern for the individual patient being recommended for treatment. For example, one participant described:

*The person may feel like, ‘Well, you’re only making me undergo treatment because you just wanna protect those around me and decrease their chance of contracting the infection, as opposed to treating me and being concerned about me.’ So I can see the person viewing the measure as ‘all but me.’* (#001)

Thus, within our interviews regarding treatment initiation, participants’ responses underscored how their decision making regarding the initiation of treatment in the context of TasP would be contingent on their ability to negotiate or ‘balance’ the risks and benefits to others (e.g., preventing transmission of the virus) with the risks and benefits to themselves (e.g., potential side effects of treatment vs. personal ‘peace of mind’). Taken as a whole, these narratives reveal that, despite having limited awareness regarding TasP (e.g., the preventative capacity of treatment; understandings about the universal availability of treatment in the Vancouver setting), participants generally responded with high levels of acceptability and receptiveness towards the approach.

### Perceived challenges and opportunities regarding long-term viral load suppression

We asked participants to reflect on the various challenges that young men who choose to initiate treatment might face with regards to achieving long-term adherence to treatment (and therefore be more likely to achieve viral load suppression). Participants described an array of different challenges, including how various social and structural conditions could serve to either positively or negatively impact young men’s capacity to adhere to a treatment regimen. For instance, a 20-year-old gay man described how the places where one lives, and their socio-economic status, would differentially influence one’s prospects for successful adherence and health outcomes in the event of an HIV diagnosis:

*Even within Vancouver, I know that like if I’m a homeless person with intravenous drug use on the Downtown Eastside* [an inner-city neighbourhood] *and I’m not eating right because I’m homeless and I don’t have a job and I’ve been mentally ill, life is going to be hard* [in the event of an HIV diagnosis]*. Whereas, if I’m a gay man on Davie Street* [Vancouver’s ‘gay village’] *with a nice middle-class job, life is going to be liveable. So I think even within Vancouver there’s like, it depends on a lot, you know, your place in society.* (#014)

A sub-set of participants who had reported a history of being street-entrenched also described how the various hardships that they experience in ‘street life’ would influence their capacity to adhere to treatment. For example, a 24-year-old straight man and a 19-year-old straight man who both were living on the street at the time of our interview described how the challenges of drug use and street life would likely influence adherence rates:

*I would think if you’re sharing rigs and out having multiple partners and all that* […] *I can’t imagine someone keeping up with the upkeep of doing that kind of treatment or something, you know?* […] *That’s just, I can’t see them having too much of a structure in their life where they would have a daily routine to do something like that.* (#031)

*Because, like, some people won’t take their pill. They might be high, they might be sleeping, they might just forget about it.* (#040)

As the interviews progressed, some participants expressed concern that individuals who choose to initiate treatment might reduce their use of other HIV prevention strategies. For example, a 23-year-old straight man described how he worried condom use would decrease among those who choose to initiate ARVs:

*Condom use would definitely go down without a doubt, just because people would be, I think people would contract HIV* […] *Like, “I can’t give somebody HIV now, so there’s no need to use a condom”. Unless they can still get them pregnant or something like that. So, I think it would go down, and I don’t think that’s a good thing.* (#029)

Some participants described how ongoing public health efforts might be able to mitigate these concerns. For example, one 23-year-old bisexual participant described how a set of tailored clinical communication strategies should be developed to inform men that TasP is not 100 % effective at preventing HIV transmission:

*I think it’s* [TasP] *good. But I think that, with it, there should be a statement that says to people that you’re not completely risk free. You are not 100 % risk free.* […] *I think there should be like, at least fail-proof prevention that comes with the medication to say that it would help protect yourself and other people, but also, however, you are not 100 % risk free so always advise your partner that you’re HIV positive.* (#038)

While most participants tended to position TasP as having an added benefit to individual HIV prevention repertoires (e.g., how young men engage with testing, treatment and prevention practices), one 20-year-old gay participant expressed frustration that, as an HIV seronegative individual, he perceived that TasP puts the responsibility of HIV prevention largely in the hands of seropositive individuals, as he described:

*As a prevention strategy, I’m like, okay, that’s focusing on the people who have HIV and could transmit it which makes sense, I guess? But as it’s like me personally as someone who would be getting the HIV, it doesn’t do anything for me.* […] *Maybe that’s just me being really self-interested in that I don’t see myself as a gay men represented there-as an HIV negative gay man.* […] *Yeah, cause it’s not ‘prevention as prevention’, it’s ‘treatment as prevention’. But it takes two people to transmit HIV, right? And* [TasP is] *only dealing with half those people.* (#014)

These findings begin to reveal how some men may ascribe complex social meanings to TasP, where it might simultaneously be perceived as over-emphasizing personal responsibility, while also being linked with risk compensation practices that have negative connotations in terms of social responsibility.

## Discussion

Overall, knowledge levels regarding ARV treatment and TasP were low. Most participants suggested that increased awareness regarding ARV treatment (including awareness of the universal availability of treatment in the Vancouver setting) would be a necessary, but not a sufficient condition to inform their decision-making regarding the use of ARVs for prevention or treatment. Instead, these decisions would more likely be contingent on their ability to negotiate or ‘balance’ the risks and benefits to themselves and others (e.g., preventing transmission of the virus; potential side effects of ARVs; personal desires to know one’s HIV status). The current study reveals how decisions to initiate TasP may be strongly influenced by both the ability to keep one’s self healthy, as well as the ability to protect others from infection. Furthermore, the findings offer insight into the complex and sometimes controversial narratives that continue to emerge regarding risk compensation practices in the context of TasP [36, 37]. This remains an area that requires more investigation to fully inform ethical approaches to implementing and scaling up TasP in ways that do not exacerbate ‘victim blaming’ [38].

The findings also highlight how the successful implementation of TasP will require a sophisticated set of clinical communication strategies-even in settings where ARVs are available universally-thereby underscoring the extent to which TasP educational and communication efforts should not be based solely at ‘target’ populations (e.g., young men), but also to those tasked with implementing TasP at the patient-clinician interface [39]. For example, clinicians require the resources and skill sets to provide knowledge that patients require to make informed decisions regarding TasP-including the side effects associated with initiating ARVs and their capacity to reduce onward disease transmission. Excellent communication strategies are particularly salient given that new evidence regarding the risks and benefits of ‘early’ initiation of treatment (i.e., immediately following seroconversion) is rapidly unfolding. Rapidly changing information ‘landscapes’ pose significant challenges to clinical discussions and demand a high level of commitment to staying current and remaining open to change as new evidence emerges regarding best clinical practice. For example, while some have expressed concern that early initiation of ARVs may lead to an increase in potential side effects, including reduced bone density and kidney damage as well as the potential for an individual’s virus to develop resistance to ARVs [40], emerging evidence indicates the individual benefits (e.g., decreased incidence of primary and secondary infections) associated with early uptake may outweigh the negative side effects [41, 42].

We were also struck by the concerns expressed by several participants that TasP strategies could tend to disregard the interests of HIV-negative individuals by not affording them enhanced agency or opportunity to engage in individual risk-reduction practices. While at first these concerns seemed to contradict the acknowledgement that TasP can have community-wide benefits by reducing the incidence of HIV, it is worth reflecting, however, the extent to which these concerns are espoused from within a socio-historical context in which public health has largely placed the responsibility of HIV prevention at the level of the individual, rather than via broad, structural and population-level interventions. Indeed, the young men in this study recognized the preventative capacity of TasP, while not privileging it as the only way to prevent HIV transmission or acquisition. In doing so, these narratives tended to align more closely with the messaging within contemporary cascades of care that emphasize combination approaches to HIV risk reduction.

Sampling (e.g., due to under-coverage, some population sub-groups of young men may not be adequately represented) and participation bias (e.g., men who choose to volunteer for a study about HIV may tend to have a similar set of experiences or beliefs about HIV) may have influenced the sample composition and do not fully reflect all variations of young men’s perspectives regarding TasP. As such, the findings are not claimed as ‘representative’ or generalizable to all young men. Moreover, the potential amplification of response biases (e.g., social desirability bias) may have also influenced the sorts of responses participants felt appropriate in the context of an interview about young men’s sexual health. While, towards the end of our data collection activities (i.e., after the 40th interview), new insights were no longer generated regarding TasP (thereby indicating theoretical saturation was attained), the findings are not claimed as ‘representative’ or generalizable to all young men. Nonetheless, our study provides rich insights into the perspectives of a diverse group of young men within Vancouver (including those from population sub-groups of men who have historically been characterized as being ‘high risk’ for HIV acquisition), thereby revealing a set of implementation challenges and opportunities for scaling up TasP programs and service delivery practices in this setting.

## Conclusion

Overall, these findings provide important opportunities for those charged with implementing and scaling up TasP efforts, including achieving the 90-90-90 testing, treatment and adherence targets. Clinicians and public health campaigners alike may benefit from better understandings of key target populations’ perspectives regarding TasP (e.g., perceptions about the availability of treatment; their perspectives on treatment initiation and long-term adherence). While findings from the current study indicate young men generally have a high receptiveness to TasP, our findings also identify key social and structural forces that will warrant ongoing consideration for TasP implementation.

## Abbreviations

ARYS: at-risk youth study
ARV: antiretorivral
CD4: cluster of differentiation antigen 4
HIV: human immunodeficiency virus
MSM: men who have sex with men
MSP: medical services plan
TasP: treatment as prevention
